# Revisiting Strephosymbolie: The Connection between Interhemispheric Transfer and Developmental Dyslexia

**DOI:** 10.3390/brainsci8040067

**Published:** 2018-04-17

**Authors:** Roberta Daini, Paola De Fabritiis, Chiara Ginocchio, Carlo Lenti, Cristina Michela Lentini, Donatella Marzorati, Maria Luisa Lorusso

**Affiliations:** 1 Department of Psychology, and Milan Center for Neuroscience (NeuroMI), Università di Milano-Bicocca, 20126 Milano, Italy; p_de_fabritiis@yahoo.co.uk (P.D.F.); ginocchio.chiara@gmail.com (C.G.); michela.lentini14@gmail.com (C.M.L.); 2 University Research Centre in Optics and Optometry, Università di Milano-Bicocca (COMiB), 20126 Milano, Italy; 3 U.O. Neuropsichiatria Dell’infanzia e Dell’adolescenza ASST Santi PaoloCarlo, 20126 Milano, Italy; carlo.lenti@unimi.it (C.L.); donatella.marzorati@libero.it (D.M.); 4 Scientific Institute IRCCS E. Medea, 22040 Bosisio Parini (LC), Italy; marialuisa.lorusso@bp.lnf.it

**Keywords:** interhemispheric transfer, developmental dyslexia, mirror writing, crossed-uncrossed difference paradigm

## Abstract

The hypothesis that an atypical hemispheric specialization is associated to developmental dyslexia (DD) is receiving renewed interest, lending some support to Orton’s theory. In this article, we investigated whether interhemispheric transfer processes (IHT) are likely to be involved in developmental dyslexia. In this study, we tested 13 children with developmental dyslexia and 13 matched controls (aged 8 to 13 years) in four different tasks. In a tactile transfer task, the dyslexic children’s performance was less accurate. In a standard Poffenberger paradigm, dyslexic children performed slower than the controls in all conditions and did not show any difference between crossed and uncrossed conditions. Furthermore, they showed an increased asymmetry of performance according to the responding hand, while controls gave more coherent responses. In a visual task of object orientation discrimination, dyslexic children had slower Response Times (RTs) than controls, especially for mirror-reversed objects in the right visual field. Finally, a higher number of dyslexic children showed mirror-drawing or mirror-writing with respect to controls. Our results as a whole show that children with DD are impaired in interhemispheric transfer, although the differences in performance among dyslexic individuals suggest the impairment of different psychophysiological mechanisms. As such, a common origin in terms of connectivity problems is proposed.

## 1. Introduction

Reading is a complex cognitive skill. On one hand, it involves fast and efficient integration of information in different modalities, i.e., visual forms and sounds. On the other hand, it recruits different cognitive abilities at once, such as perceptual integration, visual memory and phonological awareness [1,2].

Acquiring the ability to integrate and synchronize each element of this complex network takes time and practice. By 8–9 years of age, and following two years of formal teaching, a child is indeed expected to be able to read short texts, lists of words and of pseudowords fluently and quickly. When these abilities are not acquired, and in the absence of sensory deficits or low IQ, a diagnosis of developmental dyslexia (DD) may be made, according to DSM-IV-TR criteria [3], while according to the DSM-V [4] criteria, DD is included within the more general nosographic label of specific learning disorders.

Recently, a widespread agreement has grown about the fact that phonological abilities are crucial to typical and atypical development of reading skills, and recent definitions of developmental dyslexia place specific emphasis on phonology rather than on a broader range of language abilities [5]. Nonetheless, multiple deficits, including nonverbal deficits, are documented to co-occur frequently in DD, either emerging in visual tasks, or in motor and sensorimotor tasks, such as bimanual coordination, tactile proprioceptive and intermanual transfer tasks [6,7,8,9,10]. Such studies brought about scattered and far from conclusive results, which might be due to some methodological limitations [11,12,13].

The majority of those studies, indeed, test for a single nonverbal type of deficit at a time in DD samples, which vary greatly in size across studies. Criticism has also been raised as to the choice of representative experimental nonphonological tasks [14], the degree of skills required in some tasks [12,13], and the appropriate matching of controls or the adequacy of criteria for inclusion in the DD groups [15,16]. Over the last decade, research studies have therefore began using series of different experimental tasks to test different abilities within the same DD group, allowing for comparisons among deficits in different cognitive domains. Ramus and colleagues [17] analyzed adults’ performance in a wide range of tests. They concluded that the core deficit in DD is phonological, while the presence of other difficulties in nonverbal tasks might be a sign of a more severe condition, not affecting the cognitive profile of DD qualitatively. The same research group replicated the results in a developmental sample: nonphonological deficits, such as sensorimotor, for example, may be present or may not, whilst impairments in phonological processing are pervasive in DD and are alone sufficientto predict the major portion of variance in reading tests [18,19]. Menghini, Finzi, Benassi, Bolzani, Facoetti, Giovagnoli, ... & Vicari, [20] tested a wide sample of children with DD, with a wide range of experimental tasks, tapping diverse cognitive domains (spanning from reading and phonological abilities, to visuospatial perception and implicit learning abilities). The results confirmed that the most pervasive deficit in DD is a phonological one. However, salient group differences also emerged in the other non-phonological tasks, attesting to multiple, although minor, impairments in the DD cognitive profile. The authors interpreted the results as suggesting a multifactor model of impairments in DD, in agreement with those who claim a multifaceted phenomenology of dyslexia [21].

Yet, consensus with regard to the neuropsychological models accounting for such multiple behavioral deficits or subclinical performances has not been reached, so far. There is a number of popular and credited theories that have been compared (e.g., the phonological, the cerebellar, and the magnocellular theory), still two divergent hypotheses are lively contended: (1) dyslexia is a common consequence of very different causes; and (2) dyslexia is the result of dysfunction of a single “core” mechanism. In the latter hypothesis the different deficits that can be associated or not with reading difficulties could be either co-occurring but causally unrelated deficits [11,17,21] or the consequence of the same pathophysiological mechanism (e.g., impaired magnocellular neuronal development, [22]).

With regard to the nature of the deficits, we looked at one of the first theories proposed to account for DD [23]. Orton’s theory and its clearest modified version [24] pointed to abnormally lateralized functions and concomitant poor interhemispheric collaboration as possible factors leading to a number of DD symptoms. Precisely, we focused on the hypothesis that links optimal acquisition of reading abilities with an optimal interhemispheric integration as mediated by an efficient corpus callosum. This brain structure guarantees not only information transfer between the two cortices, but also that such a transfer is optimal/effective when it is timely and tuned to each within-hemisphere activity [25,26]. Consequently, on the basis of such studies, one would consider transfer speed and efficiency with respect to a number of different tasks and conditions, rather than equating efficient callosal transfer with fast transmission time.

The interhemispheric deficit theory of dyslexia has gained increasing empirical support, also thanks to anatomical studies documenting connectivity and callosal anomalies in DD) ([27,28,29,30] for a review see [31], behavioral studies about a deficit of interhemispheric transfer of tactile information in DD [7,32] and studies on individuals with congenital callosal defects, e.g., callosal agenesis, showing phonological deficits in such children [33,34]. Still, studies with multiple tasks assessing interhemispheric transfer in DD, allowing for a careful description of interhemispheric transfer profiles in DD along development, and careful investigation of how Inter-Hemispheric Transfer (IHT) may be related to reading abilities, are missing. We therefore tested the same sample of children with DD and their matched controls on a number of tasks measuring interhemispheric abilities in different modalities and at supposedly different levels of difficulty.

Time estimates of IHT (i.e., Inter-Hemispheric Transfer Times, IHTT) have been obtained by means of the Poffenberger’s paradigm. With this method, vocal or manual RTs are recorded in response to simple visual stimuli, which are presented tachistoscopically either to the left (LVF) or to the right (RVF) visual field of participants. The ipsilateral hand-visual field conditions are usually associated with faster RT compared with the contralateral hand-visual field conditions. However, the mean differences in RT between the two conditions (CUD: crossed-uncrossed difference time), are considered a reliable estimate of IHTT, provided that a sufficient number of trials is used and that attentional and spatial compatibility effects are controlled for as carefully as possible (see [15,16,17,18,19,20,21,22,23,24,25,26,27,28,29,30,31,32,33,34,35] for developmental studies; see [36,37] for clinical groups). CUDs are documented to be abnormally long in individuals with callosal defects, either congenital or acquired (whose CUD range from 96 msec to 20 msec in [38]), hinting that they are a valid measure of callosal efficiency.

Callosal efficiency can also be measured by means of tactile transfer tasks. Results with such kind of test are robust and indicate that tactile transfer improves with age, is poor in samples with callosal congenital and acquired anomalies [39], and in samples with reading and language disorders [32,40]. Positive correlations between tactile transfer and reading abilities emerge as significant only in control children, not in children with DD; conversely, in adult samples correlation between tactile transfer and reading-related abilities is salient in the dyslexic group but not in the control group [32].

The efficiency of interaction between the cerebral hemispheres can be assessed as to how it affects visual word recognition. Henderson, Barca and Ellis [41] used a paradigm based on the redundancy gain, in which reaction times or accuracy are shorter or better in detecting two identical stimuli than a single one. Henderson et al. [41] firstly compared unilateral (left and right) and bilateral visual field presentation of words and found that adults with DD did not differ from controls in processing simple words in the right visual field (left hemisphere). DD though, in contrast with controls, did not benefit from bilateral presentation, which does involve interhemispheric collaboration mediated by the corpus callosum. Participants with dyslexia performed worse than controls both in the left and bilateral visual field, with no differences between these two conditions. Further experimental conditions were devised to elucidate the precise nature of the deficit in DD. Results showed that, apart from bilateral conditions, the redundancy gain was present in dyslexic as well as in control participants, suggesting that the lack of bilateral redundancy gain in the previous experiment could be due to altered IHT in dyslexia, rather than a problem with neural summation.

Badzakova-Trajkov, Hamm and Waldie [42] administered 11 10-year-old children with phonological dyslexia and controls a redundancy gain task. They found that, in the dyslexic group, the redundancy gain for the right hand attested to a normal callosal influence (see [43] for a review on the redundancy gain effect), which was nevertheless different from controls for the left hand. Results showed an asymmetry in callosal efficiency in children with DD.

Sotozaki and Parlow [44] tested IHT abilities in teenagers by means of tasks tapping interhemispheric integration in multiple domains: they used a finger localization test requiring to reproduce the sequence of stimulated fingers, and two rhyming judgment tasks involving crossed and uncrossed visual presentation of paired words. The researchers found that the group with DD was impaired in the finger localization task, but apart from being slower in all conditions, participants with DD did not reveal impaired performance compared to controls in the crossed condition, which implies normal IHT.

The bilateral field advantage paradigm was also used in assessing IHT efficiency [6,25]. It involves the unilateral (left and right) and bilateral presentation of paired stimuli and requires to judge if coupled stimuli, such as letters, are similar or different. Judgments are required at two different levels of difficulty, and an advantage of the bilateral presentation (i.e., bilateral field advantage) emerges in the more difficult condition, while performance in the easier condition is usually faster and more accurate with unilateral presentation [45]. In a task using letters, adults with dyslexia showed bilateral field advantage likewise controls, both in accuracy and RT measures, although they were also found to be slower than controls in all conditions, i.e., unilateral and bilateral conditions (in [6]). Only in (Event-Related Potential) (ERP) measures recorded during this task did the group with Dyslexia reveal to be slower in transferring information across hemispheres, with no asymmetries in the right-to-left and left-to-right directions.

Thus far, a large amount of research shows that different paradigms testing IHT are sensitive to suboptimal performances of individuals affected by DD, in spite of differences among tasks and dyslexic individuals.

On the basis of empirical evidence and the variety of effects that a dysfunction in IHT is able to produce, it might be hypothesized that an atypical development of interhemispheric connections underlies the variability of developmental dyslexia symptoms and compromised mechanisms.

In the present study, we investigated the presence of callosal dysfunctions in children with developmental dyslexia by means of a number of different IHT tasks. Precisely, two groups, one of children with a diagnosis of developmental dyslexia and one of age-matched children with typical development, were administered four experimental IHT tasks: the standard Poffenberger’s CUD paradigm, a tactile transfer task, a lateralized orientation matching task with visual stimuli, and a writing and drawing task.

The primary aim was to ascertain IHT deficits in different tasks within the same sample of participants. As previous studies on IHT with bilateral field advantage or redundancy gain paradigms mainly employed verbal stimuli [41,46], we wanted to test whether IHT deficits can be detected beyond language-related processes. Moreover, different perceptual modalities were assessed, as tactile and visual stimuli have been administered in detection and discrimination tasks to the same group of subjects.

Multiple measures of IHT were taken in tasks where children responded to couples of visual stimuli differing—or being similar—for spatial orientation. A subsidiary aim was to explore further possible atypical asymmetries in DD as other studies did [15,42,47]. Thus, a Poffenberger’s paradigm with a reasonably high number of trials was used to replicate previous studies [6,15,35], and verify whether asymmetries in CUD emerged in the two groups of children. Finally, children were asked to draw and write common objects with both hands, as well as words and numbers. This was done in order to find a qualitative marker of delay in the development of interhemispheric communication. Fifty-two percent of healthy children aged 3–8 would spontaneously mirror write their name, while 82% and 61% of 5- and 6-year-old children, respectively, would show mirror writing at least accidentally [48]. Patients with unilateral brain injury show mirror writing and reversing of letters or numbers during writing [49,50], as well as slip-brain [51]. Samuel Torrey Orton [23] used the term *strephosymbolia* to describe the phenomenon of inversion of letters either isolated or within words, shown by dyslexic children. He interpreted this in terms of a disturbance of visual efficiency due to abnormally lateralized functions and concomitant poor interhemispheric collaboration. The concept was proposed by Bakker’s “Balance Model” of DD as well [52].

By manipulating factors and stimuli that favor left or right hemisphere processing, we hoped to gain insight into the relative contribution of each hemisphere and their collaboration efficiency.

## 2. Methods

### 2.1. Participants

From an initial group of 58 children, 26 children (6 females) were included in the present study according to selection criteria. Thirteen children had a diagnosis of developmental dyslexia (DD) and 13 matched children had normal reading ability (mean age: 10.71 for the control group and 10.58 for the group with DD; age range: 8–13). The group of children with DD was recruited via referral from San Paolo Hospital in Milan, and Association “La Prateria” for the Diagnosis and Rehabilitation of Developmental Disorders, both located in Northern Italy.

Diagnosis of dyslexia was based on the following criteria: (1) word or nonword reading speed and/or accuracy had to be at least 2 standard deviations below age mean; and (2) full-scale IQ above 85. The children who were finally included in the study were not receiving any intensive or specific reading training; comorbidity with attention deficits/hyperactivity disorders (ADHD) was excluded and they had no bilingual habits.

Reading performance was assessed by means of single word/nonword reading tests from the “Batteria per la Valutazione della Dislessia e Disortografia Evolutiva” [Battery for the assessment of Developmental Reading and Spelling Disorders] [53]. This test assesses speed and accuracy (expressed in number of errors) in reading word lists (4 lists of 24 words) and nonword lists (3 lists of 16 nonwords) and provides grade norms from the second to the last grade of junior high school. IQ was assessed through WISC-III (Wechsler Intelligence Scale-III, [54]) and scores below 85 were excluded to allow better matching of the two groups on general cognitive abilities. The control group was matched for age, gender, school grade, and handedness. Inclusion criteria for this group were the following: no reading delay (reading performance *z*-scores above 0) on word and nonword reading tests [55], no diagnosis of attention/hyperactivity disorders, no bilingual habits. Controls’ *z*-scores for speed and accuracy on word and nonword reading ranged from 0.36 to 1.03, and differed significantly from children with Dyslexia (*F*_(1,24)_ = 57.22, *p* < 0.00001, η^2^_p_ = 0.705), as expected.

Participants’ handedness was established using the Italian version of the Edinburgh manual dominance questionnaire of the test for the assessment of language [56]. All children were right-handed (mean lateralization index = 88.22% ± 14.34; 93.59% ± 10.84 for DD and control group, respectively, *F*_(1,26)_ = 1.16, *p* = 0.29).

Informed assent and consent were obtained from the children and their parents before the administration of the tests. No money was paid for participation, but at the end of the whole series of sessions, a special “degree” was awarded to the child for taking part in the project.

The study was approved by the Research Ethics Board at the University of Milan-Bicocca, as part of a major research project focusing on cognitive and communicative development of children with interhemispheric communication dysfunctions.

### 2.2. Design and Materials

Testing equipment was a PC ASUS L 3000D, with the software package E-Prime (Psychological Software Tools, Pittsburgh, PA, USA), linked to a Fujitsu Siemens visual monitor (40 inch), having a resolution of 1024 × 768 pixels, and subtending an area of 33.5 × 24.2 cm. A Wacom Intuos3 A5 graphic tablet was used to collect writing and drawing data. Noncomputerized testing was performed for tactile IHT, writing and reading. Test order was the same for all subjects.

#### 2.2.1. Interhemispheric Transfer and Integration of Tactile Information

**Figure 1:**
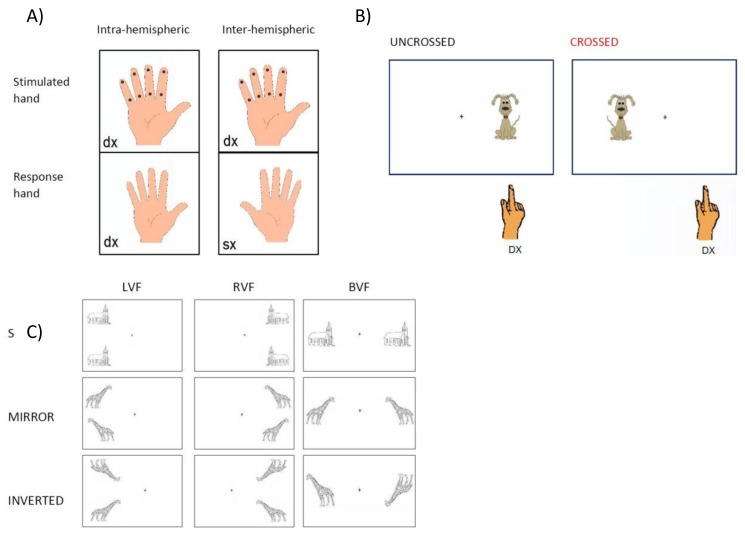
Three paradigms: (**A**) interhemispheric transfer of tactile information; (**B**) interhemispheric transfer of visual information (crossed-uncrossed differences, CUD); (**C**) same-different orientation judgment task.

Ipsi-lateral and cross-lateral somatosensory integration was assessed by means of the test developed by Volpe, Ledoux and Gazzaniga [57]. We used an abridged version of Volpe et al.’s procedure, where tactile stimulation was delivered in one single condition, which is with the hands facing palm-up. This version is the same as the one used in other studies on children with Specific Language Impairment (SLI) and Dyslexia [7,40]. During the test, the child put his/her hands resting on a table, palms up and fingers spread. The examiner used a pencil to stimulate one finger at a time. During the practice trials, the child could look at the stimulated finger, but during the test the child performed the task without visual control. After stimulation, the child was asked to indicate with the ipsi-lateral hand (uncrossed localization, 24 items for each hand) or the contralateral hand (crossed localization, 24 items for each stimulated hand) the stimulated finger and related area—distal or proximal—by touching that area with the thumb of the same hand (Figure 1A).

#### 2.2.2. Interhemispheric Transfer and Integration of Visual Information (Crossed-Uncrossed Differences, CUD)

Interhemispheric communication of lower-level processes was assessed by means of a CUD paradigm. Measures of IHTT were obtained from manual RTs in response to simple visual stimuli, presented tachistoscopically either to the left or to the right visual field of participants. The manual RT measures were based upon a previous report by Ratinckx, Brysbaert, d’Ydewalle [35] indicating that in children, likewise in adults, the difference in reaction time to a simple stimulus between the hand ipsilateral to the side of stimulation and the hand contralateral to the side of stimulation would reflect the time it took for information to cross the cerebral commissures. IHTT are considered to reliably estimate the interhemispheric transfer efficiency, provided that a sufficient number of trials is used and that attentional and spatial compatibility effects are controlled for as carefully as possible (see [15,35] for developmental studies, and Mooshagian, Iacoboni, Zaidel, 2008, 2009 for clinical groups). The latter factor was suggested to possibly account for a salient proportion of variance especially at younger ages (see [35] for comments on the application of such paradigm in developmental studies).

Stimuli were presented for 120 ms 4.0° from a central fixation point (a black cross), and depicted a small brown dog, 2.5 cm large and 4 cm high, against a white background. The task consisted of two blocks of 150 trials each: 120 lateralized stimuli (60 presented in the left visual field, and 60 presented in the right visual field), and 30 catch trials. The first block was performed responding with one hand, and the second block with the other hand (starting hand was balanced across the two groups) (Figure 1B).

The index finger of the participant’s responding hand was placed on the letter ‘b’ of an external keyboard, and was also indicated with a colored sealing-tape. As in Ratinckx et al. [35] the response button within the keyboard was aligned with the center of the computer screen, in order to control for a possible spatial stimulus–response compatibility effect.

Children were seated 57 cm from the monitor with their chin on a chin-rest. They were instructed to fixate the central black cross and were asked to press the letter ‘b’ as soon as they could detect the visual stimulus, regardless of the presentation side. They were also warned to be as quick and as accurate as possible, refraining from pressing the button when no stimuli appeared (“catch trials”).

### 2.3. Same-Different Orientation Judgment Task

A second, more complex, visual-motor task was devised to record reaction times (RTs) to tachistoscopically (150 ms) presented couples of visual stimuli. Visual stimuli were four black-and-white pictures, representing two living (giraffe, squirrel) and two nonliving objects (church, pipe), 3.5 cm large and 3 cm high. Stimuli were presented in pairs and in three possible orientations (see Figure 1C): (1) two copies of the same picture appeared in the same orientation (‘same’); (2) one copy of the picture was reflected about the vertical axis, so that the two copies of the picture were mirror-images of each other (‘mirror’); (3) one copy of the picture was reversed about the horizontal axis (‘upside-down’). Stimuli were presented on a white background; a black cross in the middle of the screen was the fixation point.

Pictures were shown in three different positions with respect to the fixation point. In the left visual field (LVF) condition, the pictures were centered 4.5° of visual angle to the left of the vertical meridian aligned with the fixation point and 5° above and below the horizontal meridian aligned with the fixation point (unilateral left visual field). In the right visual field (RVF) condition, the pictures were flashed 4.5° of visual angle to the right of the vertical meridian aligned with the fixation point, and 5° above and below the horizontal meridian aligned with the fixation point (unilateral right visual field). In the bilateral (BVF) condition, pictures were presented symmetrically one 6.5° to the left and the other 6.5° in the right of the fixation point (bilateral visual field). Such eccentricities were chosen in the attempt to keep the distance between stimuli as constant as possible, as they laid along a virtual circle with a diameter of about 6.5° and a center in the fixation point.

Subjects were seated 57 cm in front of the computer screen, and required to keep their chin on a chin-rest and to look at the central fixation point. The children were instructed to press the ‘M’ button when the stimuli were in the same orientation (‘same’) and to press the ‘N’ button when the stimuli were in a different orientation (‘upside-down’ and ‘mirror’); they were warned to be as quick as they could but also as accurate as possible. Prior to beginning practice, instructions were also given using either two small plastic bottles or pencils to demonstrate the meaning of the word ‘orientation’, asking for answers from the children until they showed they had correctly understood the task. The task was performed first with one hand, then with the other and the order was balanced across subjects.

Each child went through a training block of 10 trials and two experimental blocks of 168 trials each, with breaks about every 40 trials. In total, the experiment took about 10 min. The trials included 3 different positions, 3 different orientations, and 2 different responding hands. This resulted in a total of 56 observations per child on each visual field position (LVF, RVF and BVF), for each hand (left and right hand - LH and RH). In order to balance the ‘same’ response (due to the same condition) with the ‘different’ response (due to the upside-down and the mirror conditions), more items were added to the same condition, so that “same” responses amounted up to 43% (24 same trials), and “different” responses amounted up to 57% (16 mirror and 16 upside-down trials).

### 2.4. Drawing and Writing Task

Children were asked to draw some pictures and write some words and numbers on a Wacom graphic tablet (Wacom A5, Version Intuos 3, Publisher, Kazo, Japan), which allowed us to record either the final product or the process. Stimuli were to be drawn, with each hand, under visual control, because the first attempts to have the children draw without visual control proved to be too hard for our participants and produced less recognizable, therefore less easily analyzable, pictures. Stimuli were formed by words containing critical letters such as ‘p’, ‘q’, ‘b’ and ‘d’, like in the Italian words ‘problema’ (meaning ‘problem’), and ‘quadro’ (meaning ‘painting’), rounded objects like a clock with numbers, a visage profile, a hand and squared objects like a house and a cube. All objects had to be drawn without template. All children drew the stimuli in the same order, first with their non-dominant hand, and subsequently with their dominant hand. Drawings were coded for the orientation of the finished product (left versus right). Inter-judgment agreement among resulted to be 78% (ranging from 71% to 83%, according to the drawings considered).

### 2.5. Data Analysis

Repeated measures analyses were performed on the data, separately for each task, both on median RTs of correct responses and on accuracy, with age in months as covariate. Whenever assumptions underlying ANCOVA were not satisfied (e.g., homoschedasticity as revealed by Box’s test, homogeneity of variance across subgroups as seen by Levene’s test), we performed nonparametric analyses, as detailed in each of the following sections.

## 3. Results

### 3.1. Interhemispheric Transfer and Integration of Tactile Information

Children with dyslexia performed lower than controls both in the uncrossed and in the crossed conditions. In the crossed-hand condition, controls as well as dyslexic children made more errors. Number of errors in the uncrossed condition was very low, being null in the control group for right and left hand. Therefore, given the null variance in this subgroup in that condition, a mixed ANCOVA was performed only on the errors in the crossed conditions with hand as within factor and group as between factor.

**Figure 2:**
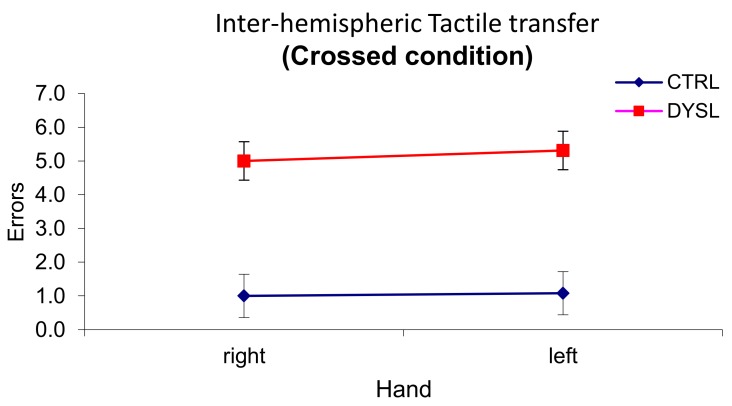
Results of the interhemispheric transfer of tactile information task.

Age in months was used as a covariate, because of the importance in our groups of the process of myelination of axons, which is considered to be completed at about 10 years of age [58,59]. Results yielded a main effect of group (*F*_(1,23)_ = 34.78, *p* = 0.000005, η^2^_p_ = 0.602), due to better performance of control with respect to dyslexic participants (mean ± sd: 1.038 ± 0.461; 5.154 ± 0.46 for control and dyslexia group respectively) (see Figure 2). As expected, a significant effect of the covariate age emerged (*F*_(1,23)_ = 7.06, *p* = 0.014, η^2^_p_ = 0.235).

### 3.2. Interhemispheric Transfer and Integration of Visual Information (Crossed-Uncrossed Differences, CUD)

#### 3.2.1. Reaction Times

**Figure 3:**
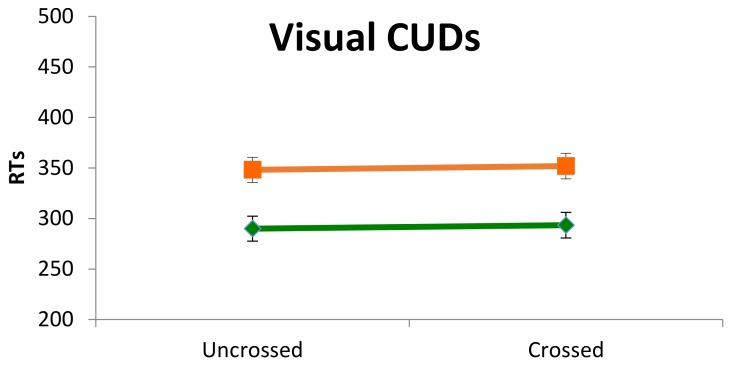
Results of the interhemispheric transfer of visual information task for each experimental group, response hand condition, and visual field condition. (the line between squares indicates participants with DD and the line between diamonds indicates control participants).

A mixed ANCOVA with Visual Field (left and right) and hand (left and right) as repeated measures, group (Control, Dyslexia) as between factor and and age in months as covariate was performed. A main effect of group was yielded (*F*_(1,23)_ = 10.736, *p* = 0.003, η^2^_p_ = 0.31), indicating that dyslexic children gave slower answers than controls (mean RT = 350.02 and 291.65, respectively), but no other significant differences emerged. Figure 3 shows the performance of controls and dyslexic participants, in each uncrossed and crossed conditions.

#### 3.2.2. Accuracy

A mixed ANCOVA with visual field (left and right) and hand (left and right) as repeated measures, group (control, dyslexia) as between factors and age in months as covariate was performed on the percentage of correct answers. The results show that dyslexic participants were less accurate than controls in all conditions (*F*_(1,23)_ = 8.53 *p* = 0.007, η^2^_p_ = 0.27), giving a mean of 95.83% ± 4.92 and 99.13% ± 1.21 of correct answers, respectively. Age turned out to be almost significant (*F*_(1,23)_ = 3.922 *p* = 0.059, η^2^_p_ = 0.1456), showing an increase of performance in both groups.

#### 3.2.3. IHTT

The lack of interaction between visual field and hand suggested that the expected difference in RT between crossed and uncrossed condition either was not present in the two samples or was covered up by a trend in two opposite directions. As several authors recommended not to collapse results across hands or visual fields because possible asymmetries in interhemispheric transfer would otherwise be confounded [35,59,60], the absence of CUD was tested separately for each hand.

CUDs for each hand and for each participant were computed, subtracting the median of RTs in the uncrossed condition from that in the crossed condition: a positive value follows a longer RT for the crossed condition, and a positive value is expected for both hands and both groups.

**Figure 4:**
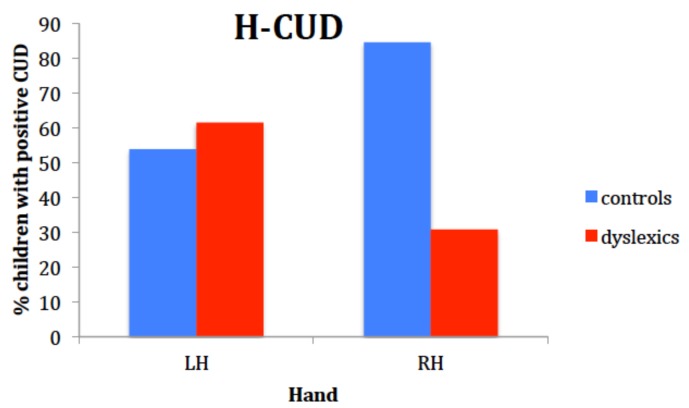
Results of the interhemispheric transfer of visual information task in terms of frequencies within the two children groups of positive CUDs.

With the left hand a similar percentage of controls and children with dyslexia had positive CUDs (53.8% vs. 61.5%, respectively, χ^2^ = 0.15, *p* = 0.50), while with the right hand, significantly more controls obtained a positive CUD than children with dyslexia (84.6% vs. 30.8%, respectively, χ^2^ = 7.72, *p* = 0.008) (Figure 4).

### 3.3. Same-Different Orientation Judgment Task

#### 3.3.1. Reaction Times

**Figure 5:**
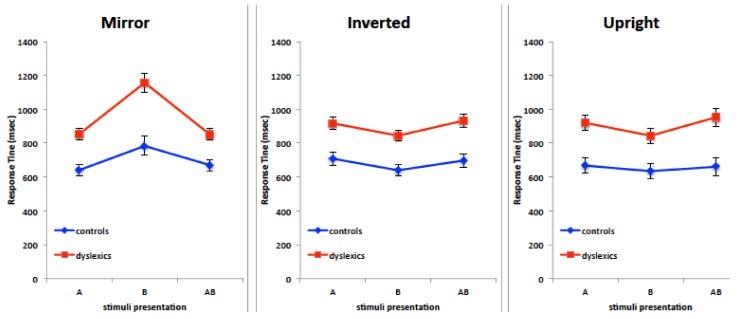
Mean response times of the orientation judgment task for the two children groups (dyslexic participants in red and controls in blue), the three visual field conditions (A: left, B: right, AB: bilateral) and the three stimulus conditions (mirror, inverted and same, respectively).

A mixed ANCOVA with hand (left and right), visual field (left, right and bilateral visual field), stimulus type (mirror, upside-down, same) as within factors, group (dyslexia, control) as a between factor and age in months as covariate, was run on median RTs. The ANCOVA yielded a main effect of group (dyslexia > control, *F*_(1,23)_ = 19.43, *p* = 0.0002, η^2^_p_ = 0.458; 996.16 vs. 755.13, respectively), a significant effect of age (*F*_(2,23)_ = 13.500, *p* = 0.001, η^2^_p_ = 0.369), and an interaction visual field by stimulus type by group (*F*_(4,92)_ = 7.977, *p* < 0.00001, η^2^_p_ = 0.257) (Figure 5).

Post-hoc comparisons (Sheffé) showed that mirror stimuli in the right visual field were dramatically difficult for the group with dyslexia, whose performance was slower compared to all the other conditions (mean RT ± sd in RVF Mirror: 1157.96 ± 49.41; average RTs in all other conditions = 889.53 ± 49.43, *p* < 0.001); whereas the other conditions did not differ from one another (*p* > 0.05). The same trend, although less pronounced, was observed in control children, with mirror stimuli taking longer than same stimuli in the right visual field only (mean ± sd: 783.88 ± 71.71; 634.58 ± 48.01; respectively, *p* = 0.04).

#### 3.3.2. Accuracy

A mixed ANCOVA with hand (left and right), visual field (left, right and bilateral visual field), stimulus type (mirror, upside-down, same) as within factors, group (dyslexia, control) as a between factor and age in months as covariate, on accuracy, showed: a main effect of group (control > dyslexia, *F*_(1,23)_ = 9.311, *p* = 0.005, η^2^_p_ = 0.288; 91.69 vs. 85.56, respectively), and an interaction between hand and stimulus type, with (*F*_(2,46)_ = 3.759, *p* < 0.03, η^2^_p_ = 0.14) and without age as a covariate (*F*_(2,46)_ = 3.03, *p* < 0.05, η^2^_p_ = 0.11). Post-hoc comparisons indicated no differences between the two hands were evident within each stimulus type, but there were more differences among conditions with the right hand (mirror differed from inverted, *p* = 0.0004; and inverted differed from same, *p* = 0.0005) than the left one (only inverted vs. same significantly differed, *p* = 0.0009).

### 3.4. Drawing and Writing Task

**Figure 6:**
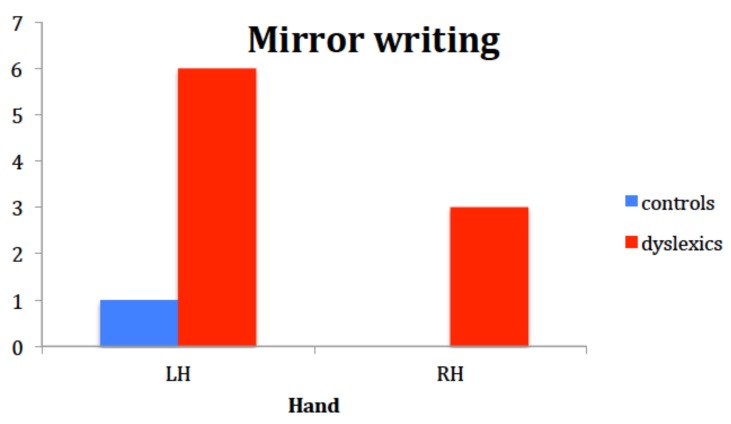
Results of the writing task in the two groups in terms of frequency of letter inversions.

Descriptive analysis on the number of inversions in writing words and drawing objects, for the left and right hand in both groups, showed that only one child in the control group mirror wrote with his left hand, while 6 out of 13 children with dyslexia did so (χ^2^ = 4.887, *p* = 0.037). With the right hand, no controls mirror-wrote, while 3 out of 13 in the dyslexia group did reverse elements of the words (χ^2^ = 3.391, *p* = 0.033). Three out of 3 children with dyslexia who mirror-wrote with the right hand, mirror-wrote with the left hand as well. As frequency of mirror writers was so small, and so was the number of inversion, no ANOVA could be performed to test the effect of the group and age. However, a careful look at age was paid. The only control who did reversals with his left hand was 109 months-old. Coherently, children with DD who did not mirror write had a mean age of 127 months ±14 months. These data suggest that mirror writing tends to disappear with age but still it is more frequent in the DD group than in the control group (Figure 6).

### 3.5. Correlations

In order to support the association between IHT efficiency and reading ability, we performed a correlation analysis between a series of IHT measures and a series of reading tests.

Only those IHT measures that resulted significantly different between the two groups were considered, which are the number of errors in the crossed condition of tactile information task, the speed of detection in the crossed condition of visual information task, the speed and accuracy of visual discrimination in the mirror condition in the right visual field of object orientation task.

First of all, Pearson’s correlations were computed in the whole group of participants. As a second step, correlations were computed separately for the two groups and it was ensured that the correlations emerging from the first analysis would show comparable coefficients in the group with (and, secondarily, without) dyslexia (independently of *p*, which is highly penalized by the low number of participants in each group).

In order to obtain more reliable indicators of reading ability in spite of the low number of participants, a “global” score was computed by averaging speed and accuracy *z*-scores for word and nonword reading into a single score. In addition, separate correlations with speed and accuracy *z*-scores in word and nonword reading were analyzed in order to have a more detailed picture of the relationships between IHT and specific reading processes. No correction for multiple comparisons was applied considering the high correlations existing among the variables belonging to the same category (either IHT or reading). However, only correlation coefficients larger than 0.4 in the whole sample and larger than 0.5 in each group were considered.

Table 1 summarizes the results. It can be seen that the global reading score correlated with all IHT measures (positively with accuracy, negatively with errors or speed) except for accuracy in visual discrimination in the mirror condition in the RVF. More specifically, speed measures in both word and nonword reading correlated with all IHT measures (except for accuracy in mirror condition in the RFV), while accuracy in word and nonword reading correlated with speed in visual discrimination in the mirror condition in the RVF, and accuracy in word reading also correlated with tactile interhemispheric transfer.

These correlations were partially confirmed in the group of dyslexic children only (Table 2): the global reading *z*-score confirmed its correlation with speed in visual discrimination in the mirror condition in the RVF. Most notably, new correlations emerged in the dyslexic group that were clearly compensated and cancelled out by opposite correlations in the control group. Specifically, accuracy in nonword reading correlated with the CUD speed parameter, suggesting a role of visual IHT speed in the indirect decoding route for reading in dyslexia, and striking correlations of opposite sign appeared to link accuracy in the mirror condition in the RFV with speed in word and nonword reading. 

Finally, mirror writing was significantly correlated with speed in both words and nonwords reading in the whole sample, and with accuracy in nonword reading in the DD group (Table 3).

## 4. Discussion

The present study investigated IHT and hemispheric asymmetries by administering four different tasks to a sample of children with DD and typical development. . Results showed suboptimal and atypical performance of the DD group in all tests. Precisely, in the tactile transfer test, children with DD revealed to be (frankly) impaired: they produced many more errors than controls especially in the crossed condition, which requires interhemispheric transfer. Such an impairment in the tactile modality confirmed several previous results with both children and adults with DD [7,32]. The fact that Age was significant as a covariate may suggest that all children improve in IHT ability along development, however DD children’s difficulties remain clinically significant. Such developmental trajectories would need confirmation in further studies.

Like in previous studies with tactile transfer tests, no differences between hands emerged, therefore difficulties in the interhemispheric communication of tactile information seemed equal in both directions.

Results in the classic Poffenberger’s paradigm showed that children with DD were generally slower and less accurate than controls, but they did not reveal to be specifically impaired in the crossed condition. These behavioral and rather generic features of children with DD have been reported by other researchers [61]. Our results with this paradigm, though, are not unequivocal because the interaction hand by visual field was not statistically significant and, according to Davidson et al. [15] and Marzi et al. [60], the interaction is a robust finding and a sort of a prerequisite in order for the data to be interpreted within the paradigm rationale. However, qualitative, non-parametric analyses were performed which suggested that asymmetry of IHT directions is the same as in Davidson et al. [15]: time estimates of IHT are faster from right to left in the DD sample, as it was shown by the higher number of DD children with negative CUD for the right hand. In fact, the percentage of positive CUD with the right hand for the controls in our study was even higher than in Davidson et al.’s sample (86% vs. 50% respectively), while the percentage of dyslexic children having a positive CUD with the right hand was very similar (30% vs. 36%). It looked like if, especially children with DD, did not benefit from processing the visual stimuli in the left hemisphere and elaborating a motor response within that hemisphere. Suboptimal functioning of the left hemisphere in DD was also reported by Broman et al. [61], who described a very poor performance in the right hand-right visual field condition with the same paradigm.

Results in the orientation judgment task showed that mirror stimuli are processed with a remarkable difficulty in the right visual field/left hemisphere condition. Admittedly, these results were unexpected for two main reasons. On the basis of Corballis et al.’s work on mirror images perception, a bilateral disadvantage could be hypothesized because of interhemispheric exchange of mirror-reversed images subsequent to callosal cross-cortical homotopic projections [24]. Also, the left hemisphere is credited with residual spatial abilities tied to categorical spatial representation, such as above-below, left-right, involving symbolic descriptions of the relations between objects. Consequently, the left hemisphere was not expected to be impaired at such discrimination with nonverbal visual stimuli in the dyslexic group. The peculiar difficulty in processing mirror stimuli in the RVF was exhibited also by controls, but RTs in their case appeared less different from RTs in the other VFs.

Somehow paradoxically, a similar difficulty in processing mirror images in the right visual field/left hemisphere was reported by Funnel and colleagues [62] and by Corballis and colleagues [51] in three split-brain participants. The JV patient was administered a number of tests with visual objects to discriminate, either nameable or not, rich or poor in details: in all tests the patient exhibited a clear impairment of left hemisphere at judging mirror objects. Results by DDV and DDC, i.e., the other two split-brain patients, confirmed such a LVF superiority at judging left-right orientation of visual stimuli, which was present, although less remarkably, in adult controls as well. Therefore, looking at the poor results of our participants in the RVF with mirror stimuli, one could notice a resemblance with the poor performance by split-brain patients. It seemed to be possible that the right hemisphere can better process mirror-reversed images, and that the left hemisphere transfers such stimuli to the right hemisphere. Whenever such transfer is impeded, like in split-brains, or defective, like in children with DD, the performance in the RVF is shown to be very poor.

Children’s performance in the writing and drawing task showed that the group with DD was more prone to write mirror-reverse letters and numbers than controls, especially when using the left hand. Although mirror writing is described in the literature to occur mainly with the left, non-dominant hand, results seemed incoherent with the idea that the right hemisphere is in charge for mirror discrimination. In fact, half of our mirror-writers did mirror-writing with their right hand too. This could suggest that, in children with DD, mirror writing, though a sporadic and rare phenomenon, is not dependent on peripheral factors such as the hand used to draw, but rather on a representational problem with respect to left-right orientation.

The correlations between IHT measures and reading indices support a relationship between the two in the group with dyslexia. These results further corroborate the claim that our results depend on the role of the corpus callosum in building the representation of a stimulus in each hemisphere. *Strephosymbolie* refers to this phenomenon of mirror writing and retrieves Orton’s theory on the relationship among corpus callosum, specular representations of visual stimuli in the two hemispheres and developmental dyslexia.

The correlation of the frequency of mirror writing and reading measures offers interesting insights into the role of callosal transfer for reading and writing. Indeed, mirror writing in the whole group of participants appears to correlate with speed of reading for both words and nonwords. By contrast, an interesting correlation emerges in the group of participants with dyslexia between mirror writing and accuracy of nonword reading. Again, this correlation suggests a connection between mirror writing and accuracy in visual sequential decoding (as needed to read nonwords accurately) which strictly reminds of the concept of *strephosymbolie* as proposed by Orton: confusion between the two representations of the word in the two hemispheres has to do with the ability to read, especially involving the indirect route for reading.

In conclusion, by using different modalities and different tasks, we found evidence that interhemispheric transfer processes (IHT) are likely to be involved in developmental dyslexia. The finding that measures of IHT were affected by Age, suggests that the difficulty could be even larger in younger kids and present at the very beginning.

A speculative reasoning could be that a delay in the development of interhemispheric connections could be the pathophysiological mechanism underlying the general delay in cognitive functions [63] and in temporal processing [22] described as the cause of various visuoperceptual, attentional and phonological difficulties.

A clear limitation of this study is the small size of the sample. Another one is that we did not measure the influence of attention on response times, although attention cannot explain some specific effects.

Future research should study the development of IHT with larger sample size, in different cohorts of readers with typical development and with dyslexia in order to identify the most critical periods to exploit and the most critical tasks for the design of more effective and more selectively directed intervention programs.

## Figures and Tables

**Table 1:** Correlations between IHT measures (in columns) and reading indices (rows) in the whole sample (26 children). Correlation coefficients larger than 0.4 are highlighted in bold.

Word and Nonword Reading Tests (Zoccolotti et al., 2005)	Tactile Information Task-Crossed Condition	Visual CUD: Speed-Crossed Condition	Visual CUD: Accuracy-Crossed Condition	Orientation Judgment Task: Speed-Mirror Condition RVF	Orientation Judgment Task: Accuracy-Mirror Condition RVF
Mean *Z* score	**−0.523**	**−0.4502**	**0.4181**	**−0.6825**	0.1787
	*p* = 0.006	*p* = 0.021	*p* = 0.034	*p* < 0.001	*p* = 0.382
Nonword Speed *Z* score	**−0.4265**	**−0.567**	**0.427**	**−0.6163**	0.1233
	*p* = 0.030	*p* = 0.003	*p* = 0.030	*p* = 0.001	*p* = 0.548
Word Speed *Z* score	**−0.488**	**−0.4369**	**0.4135**	**−0.6333**	0.1304
	*p* = 0.011	*p* = 0.026	*p* = 0.036	*p* = 0.001	*p* = 0.525
Nonword Accuracy *Z* score	−0.3535	−0.1166	0.1936	−0.4292	0.1751
	*p* = 0.076	*p* = 0.571	*p* = 0.343	*p* = 0.029	*p* = 0.392
Word Accuracy *Z* score	**−0.4473**	−0.3467	0.3234	**−0.5692**	0.1849
	*p* = 0.022	*p* = 0.083	*p* = 0.107	*p* = 0.002	*p* = 0.366

**Table 2:** Correlations between IHT measures (in columns) and reading indices (rows) in the group with DD (13 children). Correlation coefficients larger than 0.5 are highlighted in bold.

Word and Nonword Reading Tests (Zoccolotti et al., 2005)	Tactile Information Task-Crossed Condition	Visual CUD: Speed-Crossed Condition	Visual CUD: Accuracy-Crossed Condition	Orientation Judgment Task: Speed-Mirror Condition RVF	Orientation Judgment Task: Accuracy-Mirror Condition RVF
Mean *Z* score	0.3883	0.2363	−0.0802	**−0.5029**	**−0.5595**
	*p* = 0.190	*p* = 0.437	*p* = 0.795	*p* = 0.080	*p* = 0.047
Nonword Speed *Z* score	0.3702	−0.2983	0.0743	−0.2336	**−0.5394**
	*p* = 0.213	*p* = 0.322	*p* = 0.809	*p* = 0.443	*p* = 0.057
Word Speed *Z* score	0.1776	0.0573	0.0596	−0.3781	−0.4413
	*p* = 0.562	*p* = 0.852	*p* = 0.847	*p* = 0.203	*p* = 0.131
Nonword Accuracy *Z* score	0.1885	**0.5298**	−0.2257	−0.1426	−0.104
	*p* = 0.537	*p* = 0.063	*p* = 0.458	*p* = 0.642	*p* = 0.735
Word Accuracy *Z* score	0.2607	0.2323	−0.1412	−0.3484	−0.233
	*p* = 0.390	*p* = 0.445	*p* = 0.645	*p* = 0.243	*p* = 0.444

**Table 3:** Correlations between mirror writing and reading indices (rows) in three groups: all 26 children, 13 with DD, 13 controls. Correlation coefficients larger than 0.5 in the whole group and 0.4 in single groups are highlighted in bold.

Word and Nonword Reading Tests (Zoccolotti et al., 2005)	Mirror Writing 26 Children	Mirror Writing 13 DD Children	Mirror Writing 13 Controls
Mean *Z* score	−0.364	−0.216	0.178
	*p* = 0.068	*p* = 0.479	*p* = 0.56
Nonword Speed *Z* score	**−0.410**	−0.362	0.045
	*p* = 0.038	*p* = 0.224	*p* = 0.885
Word Speed *Z* score	**0.427**	−0.354	0.0
	*p* = 0.03	*p* = 0.236	*p* = 1
Nonword Accuracy *Z* score	−0.018	**−0.572**	0.357
	*p* = 0.932	*p* = 0.041	*p* = 0.231
Word Accuracy *Z* score	−0.183	−0.354	0.267
	*p* = 0.37	*p* = 0.236	*p* = 0.377
